# Metagenomic deep sequencing reveals association of microbiome signature with functional biases in bovine mastitis

**DOI:** 10.1038/s41598-019-49468-4

**Published:** 2019-09-19

**Authors:** M. Nazmul Hoque, Arif Istiaq, Rebecca A. Clement, Munawar Sultana, Keith A. Crandall, Amam Zonaed Siddiki, M. Anwar Hossain

**Affiliations:** 1 The Laboratory of Microbial Genetics and Bioinformatics, Department of Microbiology, University of Dhaka, Dhaka, 1000 Bangladesh; 2 Department of Gynecology, Obstetrics and Reproductive Health, Faculty of Veterinary Medicine and Animal Science, Bangabandhu Sheikh Mujibur Rahman Agricultural University, Gazipur-1706, Bangladesh; 3 Computational Biology Institute, Department of Biostatistics and Bioinformatics, Milken Institute School of Public Health, the George Washington University, Washington, USA; 4 Department of Pathology and Parasitology, Chittagong Veterinary and Animal Sciences University, Chittagong-4202, Bangladesh; 5 Department of Developmental Neurobiology, Graduate School of Medical Sciences, Kumamoto University, Kumamoto, Japan; 6 Present Address: Vice-Chancellor, Jashore University of Science and Technology, Jashore 7408, Bangladesh

**Keywords:** Infection, Microbiome

## Abstract

Milk microbiomes significantly influence the pathophysiology of bovine mastitis. To assess the association between microbiome diversity and bovine mastitis, we compared the microbiome of clinical mastitis (CM, n = 14) and healthy (H, n = 7) milk samples through deep whole metagenome sequencing (WMS). A total of 483.38 million reads generated from both metagenomes were analyzed through PathoScope (PS) and MG-RAST (MR), and mapped to 380 bacterial, 56 archaeal, and 39 viral genomes. We observed distinct shifts and differences in abundance between the microbiome of CM and H milk in phyla *Proteobacteria*, *Bacteroidetes*, *Firmicutes* and *Actinobacteria* with an inclusion of 68.04% previously unreported and/or opportunistic strains in CM milk. PS identified 363 and 146 bacterial strains in CM and H milk samples respectively, and MR detected 356 and 251 bacterial genera respectively. Of the identified taxa, 29.51% of strains and 63.80% of genera were shared between both metagenomes. Additionally, 14 archaeal and 14 viral genera were found to be solely associated with CM. Functional annotation of metagenomic sequences identified several metabolic pathways related to bacterial colonization, proliferation, chemotaxis and invasion, immune-diseases, oxidative stress, regulation and cell signaling, phage and prophases, antibiotic and heavy metal resistance that might be associated with CM. Our WMS study provides conclusive data on milk microbiome diversity associated with bovine CM and its role in udder health.

## Introduction

Mastitis is one of the most prevalent diseases in the dairy industry with the highest clinical and economic significance worldwide^1^. The condition usually happens when pathogenic microbes enter the mammary gland, mostly by the disruption of the physical barriers of the mammary quarters, requiring prompt and appropriate host defenses to prevent colonization and subsequent disease pathology^2^. Diverse groups of microbes are known to colonize the mammary quarters of cows and have evolved novel mechanisms that facilitate their proliferation, leading to clinical mastitis (CM). Despite knowledge of a few of these invading microbial groups, the etiology of bovine mastitis is continuously changing, with new microbial species identified as causing disease frequently. Additionally, although bacteria are the main cause of mastitis^3^, other microbes like archaea, viruses, and fungi might be associated with the disease process^4^ and should therefore be investigated as well. During the progression of the mastitis, dysbiosis of the milk microbiome can occur with the increase of opportunistic pathogenic bacteria and reduction of healthy commensal bacteria^5^. Until recently, investigations of the microbiome associated with bovine mastitis have been mostly restricted to individual pathogen isolation and characterization. The disease is caused by epidemiologically diverse groups of microorganisms and categorized into contagious and environmental mastitis^6^. The udder of the dairy cows is the primary reservoir of contagious pathogens including *Staphylococcus aureus*, *Streptococcus agalactiae*, *Streptococcus dysgalactiae*, *Mycoplasma* spp., and *Corynebacterium bovis*^1,6^. The involvement of the bovine mammary gland microbiome in the host-pathogen interaction has infrequently been investigated except during the infectious episode^7^. Environmental pathogens such as *Escherichia coli*, *Klebsiella pneumoniae*, *Klebsiella oxytoca*, *Enterobacter aerogenes*, *Streptococcus dysgalactiae*, and *Streptococcus uberis*^1,6^ can also be implicated in disease.

Rapid advances in high-throughput NGS technology and bioinformatics tools^8^ during the last decade have initiated a transition from clinical microbiology to genomic characterization of the microbiome associated with infection, including mastitis which affects lactating women^5^ and animals^9^. However, until recently, 16S rRNA partial gene sequencing approach remained the most commonly used genomic survey tool in studying the bovine mastitis microbiome^1,7^. This technique is highly useful in resolving more than 90.0% of isolates at the genus level. However, it has a number of inherent limitations including the polymerase chain reaction (PCR) bias, inability to detect viruses, lower taxonomic resolution at the species or strain level, and limiting information on gene abundance and functional profiling^10^. These factors eventually limit the ability to fully explore the microbiome and its interaction with the host comprehensively. A complimentary, shotgun whole metagenome sequencing (WMS) approach reflecting the total microbial makeup of a sample (bacterial, archaeal, fungal, viral) has been used successfully to gain insights into the phylogenetic composition and species diversity of a variety of microbiomes^11^, including profiling of their functional attributes^12^. Thus, data such as the identity and abundance of genes related to microbial metabolism, virulence, and antibiotic resistance can be generated simultaneously enabling identification of unknown etiological agents that play a role in mammary gland pathogenesis.

The relatively overexpressed genes associated with immune suppression^13^, systemic oxidative stress^3^ and inflammatory processes^14^ coming from metabolic activities of the microbiome (bacteria, archaea and virus) are crucial factors for the development and progression of bovine CM. Surprisingly from the beginning of the twenty-first century, a rapid increase in antimicrobial resistance, particularly multidrug resistance (MDR), in bovine mastitis pathogens has been observed, which corresponds with the relatively higher abundance of genes coding for antibiotics and toxic compounds resistance in the CM milk microbiome^15^. Therefore, summarizing the variation in biota and protein functional diversity in clinical and healthy milk microbiomes using cutting-edge genomic technologies like WMS^16^ and associated bioinformatic tools is essential in understanding the pathophysiological conditions of bovine CM. Here we report the first study to apply high-throughput sequencing data (on an average 23.01 million reads per sample) to investigate the microbiome of bovine CM and H milk^17^. The results revealed that cows suffering from CM have a distinct microbial community with altered protein functions compared to their healthy counterparts, which leads to pathophysiological conditions.

## Results

### Structure and composition of the bovine milk microbiome

The rarefaction curves based on observed species metrics reached the plateau after on average 23.01 million reads (Supplementary Fig. 1a; Data 1) suggesting that the depth of coverage was sufficient to capture the entire microbial diversity within the samples. We found significant differences in alpha-diversity (Observed species and Shannon estimated) between the clinical mastitis (CM) samples and healthy controls (H) regardless of the method used to tabulate microbial abundances i.e., either PathoScope (PS) or MG-RAST (MR) (PS; *p* = 0.005, MR; *p* = 0.007, U test), showing higher diversity in the microbial ecosystem of CM milk (Supplementary Fig. 1b–d). Beta diversity (PCoA) also showed significant microbial disparity (*p* = 0.001) between CM and H sample groups (Supplementary Fig. 2a,b). At phylum level, NMDS also showed distinct differences (*p* = 0.001) between the sample categories (Supplementary Fig. 3a,b).

**Figure 1:**
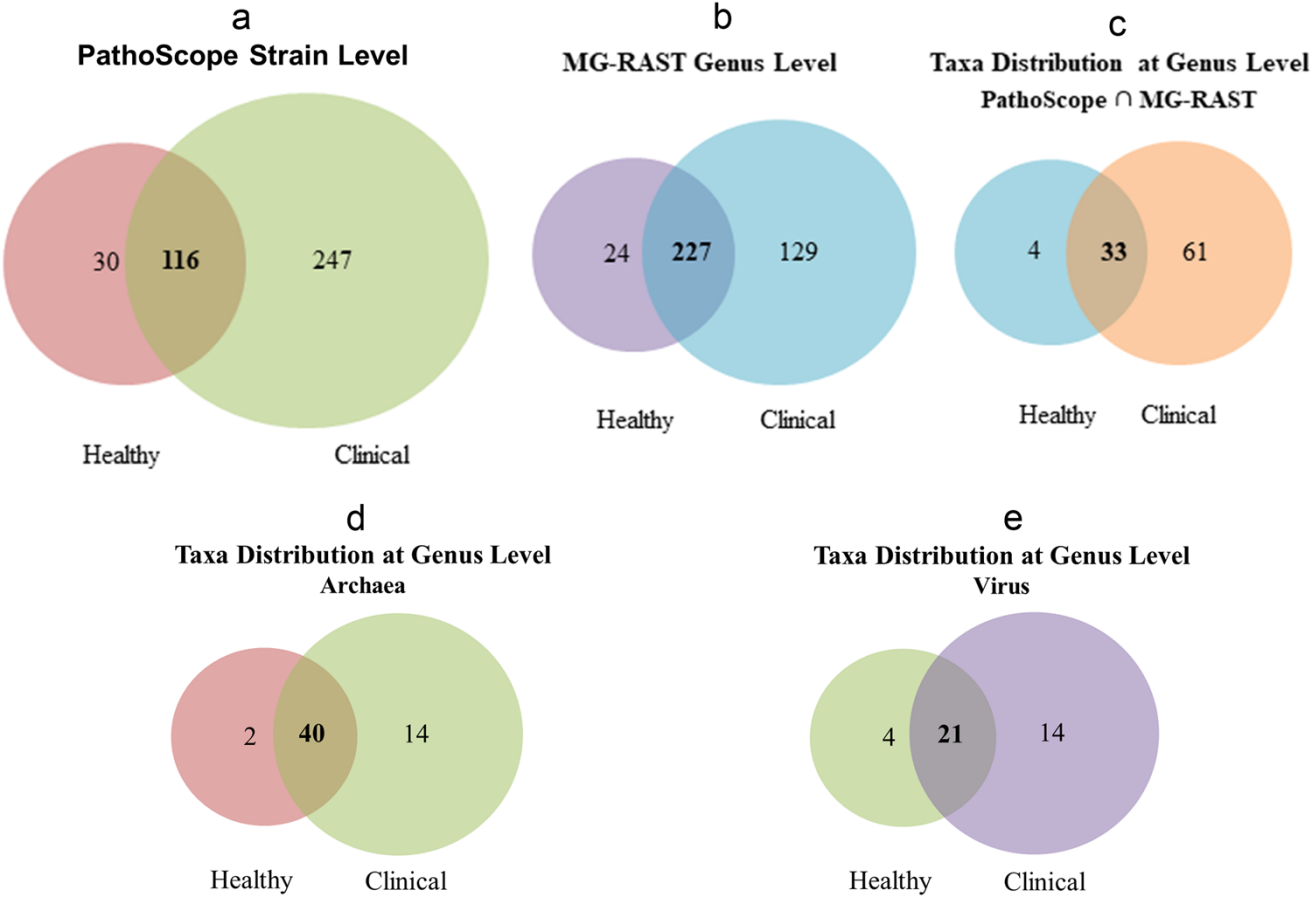
Taxonomic composition of bovine milk microbiome. Venn diagrams representing the core unique and shared microbiomes of bovine clinical mastitis (CM) and healthy (H) milk. (**a**) Venn diagram comparison of bacteria at strain level by PathoScope (PS), (**b**) Venn diagram showing unique and shared bacterial genera by MG-RAST (MR), (**c**) Shared and unique bacterial genera distribution between PS and MR, (**d**,**e**) Venn diagrams representing unique and shared archaeal and viral genera, respectively found in bovine milk as analysed with MR pipeline. Microbiota shared between the conditions are indicated in bold.

At the domain level, bacteria were the most abundant community, with an average abundance of 99.49%, followed by viruses (0.38%), and archaea (0.13%) (Supplementary Data 1). Though the relative abundance of microbes was higher in CM compared to H milk, the abundance fluctuated more (CV = 886.90 vs 511.80; PS, CV = 1521.41 vs 1221.92; MR). The unique and shared distribution of microbial taxa found in CM and H samples by the two analytic tools is represented in Venn diagrams (Fig. 1). A total of 363 bacterial strains in CM and 146 in H metagenomes were detected in PS analysis, of which 116 (29.51%) strains were present in the both sample sets (Fig. 1a). However, with the MR pipeline, 356 and 251 bacterial genera were detected in CM and H samples, respectively whereas 227 (63.8%) genera were common in both metagenomes (Fig. 1b). By comparing the detected bacterial genera between two analytic tools, 98 unique genera were identified, of them 62.24% genera were solely associated with the onset of bovine CM (Fig. 1c; Supplementary Data 2). In addition, MR detected 54 and 42 archaeal, and 35 and 25 viral genera in CM and H samples, respectively, and among them 25.00% and 35.00% archaeal and viral genera, respectively had sole association with CM (Fig. 1d,e). Unlike MR, PS detected only one archaeal genera (*Methanobrevibacter*) in CM and none in H samples.

### CM-associated bacteria changes at the genus level

**Figure 2:**
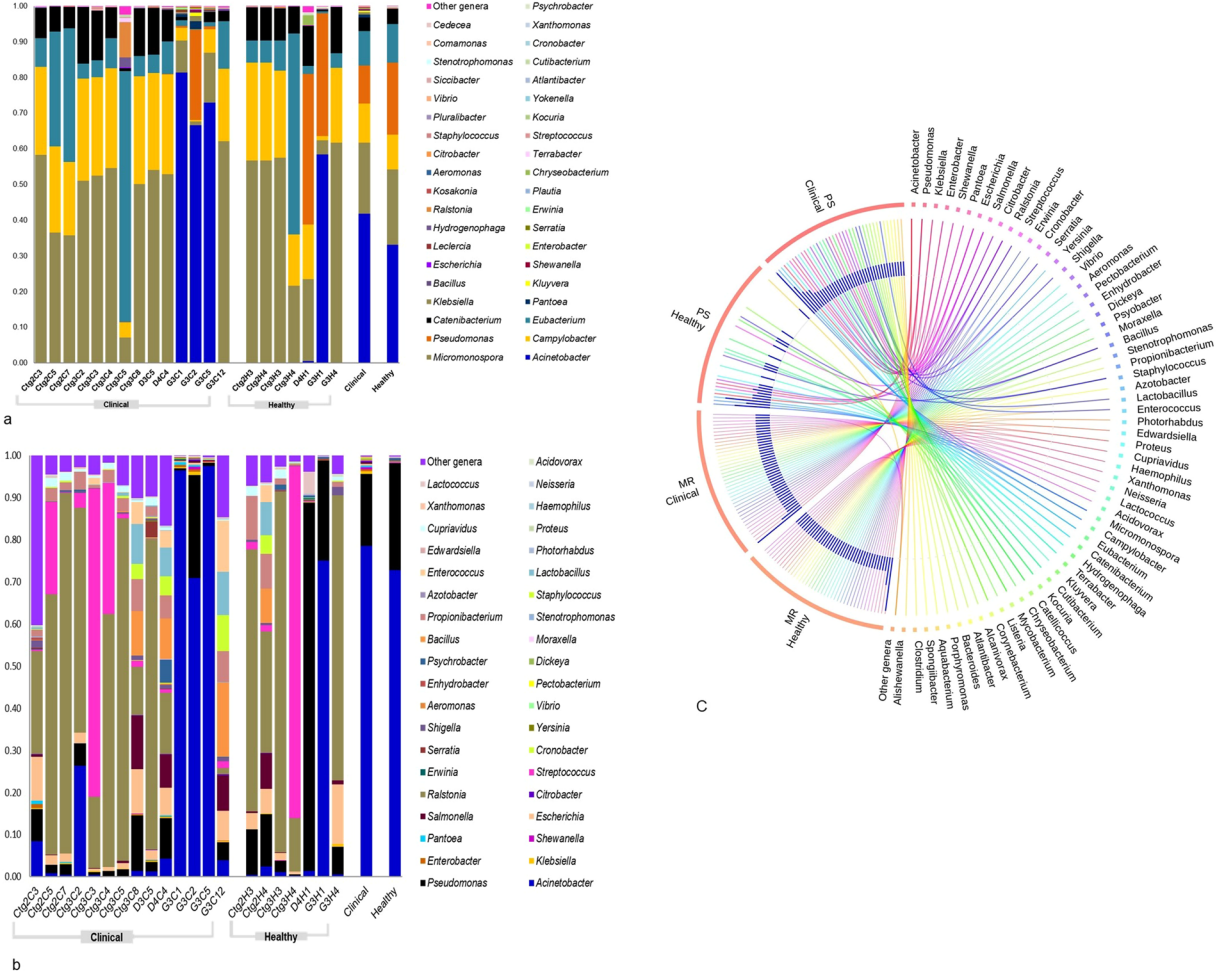
Taxonomic profile of 40 most abundant bacterial genera in bovine clinical mastitis (CM) and healthy (H) milk samples. (**a**) Relative abundance through PathoScope (PS) and (**b**) relative abundance through MG-RAST (MR) analyses. The 39 most abundant bacterial genera are sorted by descending order of the relative abundance in 21samples, with the remaining genera grouped into the ‘Other genera’. Each stacked bar plot represents the abundance of bacteria in each sample of the corresponding category, where the last two bar plots depict overall relative abundance of bacterial genera between CM and H samples, respectively. (**c**) The circular plot illustrates the relative abundance of the top 40 bacterial genera in CM and H milk samples analysed through PS and MR. Taxa in both metagenomes are represented by different colored ribbons in both tools. The relative abundancies are illustrated by the sizes of each color segment in the outer circle and the inner blue colored bars. Part of the microbiome is shared by both sample categories (CM-H milk) and part is analytic tool specific (PS-MR). Notable differences between the bacterial populations are those where the taxon is abundant in CM samples and effectively undetected in the H milk. Sample names: suffix ending in C refers to clinical (CM) and that ending with H refers to healthy (H) milk samples.

The current microbiome study demonstrated notable differences among the microbial community in CM and H milk using both bioinformatics tools. *Proteobacteri*a, *Bacteroidetes*, *Firmicutes*, and *Actinobacteria* (contributing to 96.51% of the total sequences, U test, *p* = 0.001) were the four most abundant phyla in PS and MR analyses. The relative abundances of the top 40 bacterial genera were compared between the CM and H cohorts through PS and MR analyses (Fig. 2). Among the predominating phyla,* Proteobacteria* was the most diverse and included a wide variety of genera including *Acinetobacter*, *Pseudomonas*, *Escherichia*, *Vibrio*, *Erwinia*, and *Pantoea*. The phylum *Firmicutes* was dominated by *Streptococcus*, *Enterococcus*, *Staphylococcus*, and *Bacillus* while *Chryseobacterium*, *Porphyromonas* and *Prevotella* were predominating in *Bacteroidetes* phylum, and *Corynebacterium* was the most abundant genus in the phylum *Actinobacteria*. Among the detected genera, *Acinetobacter* (60.14%), *Campylobacter* (10.93%), *Pantoea* (0.66%), *Klebsiella* (0.63%), *Kluyvera* (0.42%), *Salmonella* (0.31%), *Enterobacter* (0.30%), *Shewanella* (0.30%), *Escherichia* (0.28%), *Citrobacter* (0.15%), and *Bacillus* (0.10%) had higher mean relative abundance in CM samples regardless of analytical tool, while the rest of the genera had relatively lower mean abundances (<0.10%). In contrast, the H milk metagenomes also had higher mean relative abundances of *Acinetobacter* (52.90%) in PS and MR pipelines followed by *Pseudomonas* (22.81%), *Micromonospora* (10.57%), *Eubacterium* (5.37%), *Catenibacterium* (2.12%), and *Ralstonia* (0.12%), and the rest of the genera had much lower abundances (<0.10%). In general, MR detected higher numbers of microbial genera than PS (Supplementary Tables 2 and 3), however results from the both tools were concordant, with 98.00% of the total microbial abundance composed of shared genera (Supplementary Table 4; Data 2).

### CM-associated bacteria changes at the strain level

**Figure 3:**
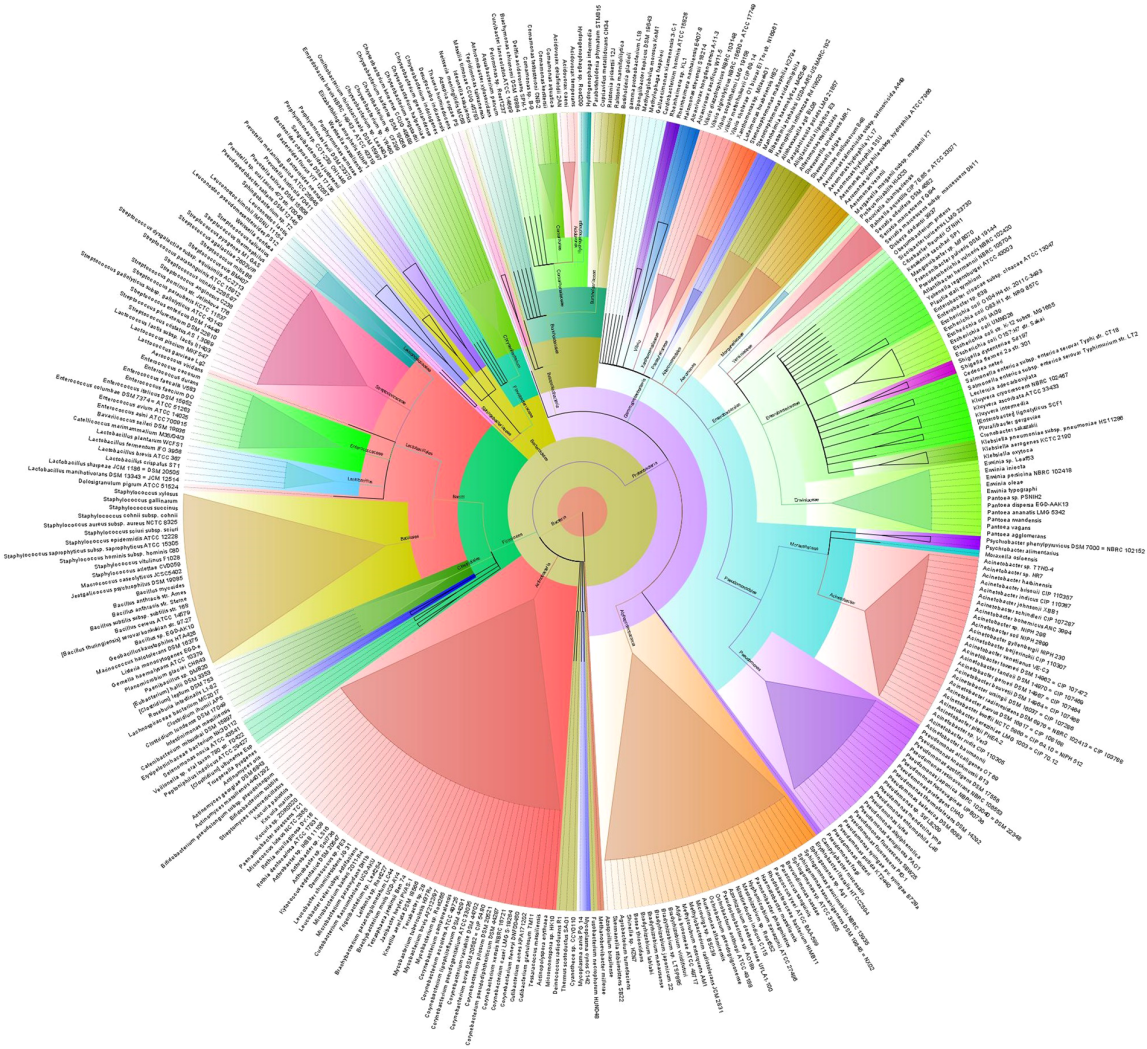
The species and/or strain level taxonomic profile microbiota associated with bovine clinical mastitis (CM). Sequences are assigned to different taxonomic index in PathoScope (PS) analysis using minimum identity of 95% and minimum alignment length 20 as cutoff parameters and the circular phylogenetic tree is constructed based on the neighbor-joining algorithm using FigTree. The round tree illustrates 363 unique strains of bacteria in CM milk metagenomes. The inner circle represents the root of the microbiome defined as bacteria present in all samples. The outer circles represent different strains of bacteria is defined as species (with different strains) present in >50% of samples of the corresponding groups. For the outer circles, the width of a segment is proportional to the observed incidence for that species. Different colors are assigned according to the taxonomic ranks of the bacteria. The species and/or strains in the phylogenetic tree are also available in Supplementary Data 2.

**Figure 4:**
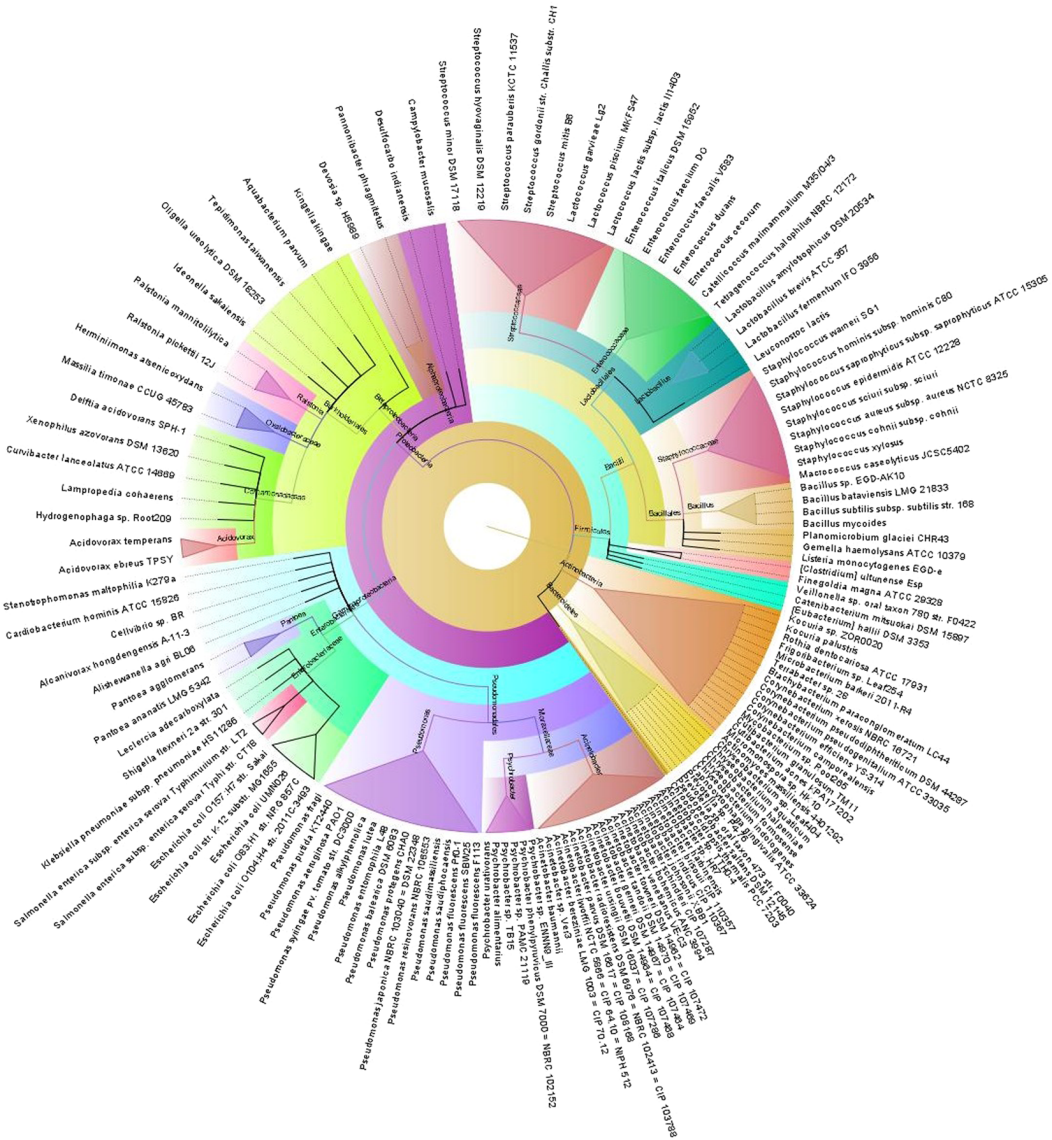
The species and/or strain level taxonomic representation of microbiome in bovine healthy (H) milk samples. Sequences are assigned to different taxonomic index in PathoScope (PS) analysis using minimum identity of 95% and minimum alignment length 20 as cutoff parameters and the circular phylogenetic tree is constructed based on the neighbor-joining algorithm using FigTree. The round tree illustrates 146 unique strains of bacteria in H milk metagenomes. The inner circle represents the root of the microbiome defined as bacteria present in all samples. The outer circles represent different strains of bacteria is defined as species (with different strains) present in >50% of samples of the corresponding groups. For the outer circles, the width of a segment is proportional to the observed incidence for that species. Different colors are assigned according to the taxonomic ranks of the bacteria. The species and/or strains in the phylogenetic tree are also available in Supplementary Data 2.

We further investigated whether strain level relative abundances of the bacteria differed between CM and H samples (Figs 3 and 4). The CM milk metagenome had significantly (*p* = 0.001) higher number of bacterial strains than the H milk, and among the detected strains, 62.85% had unique association with bovine CM, and 7.63% were solely found in H milk (Fig. 1a). The presence of few predominating bacterial species in both categories of samples suggests that the crucial differences might be occurring at the strain level, and most of the species identified in each sample were represented by a single strain. The CM milk metagenome was dominated by 26 strains (7.16%) of *Acinetobacter* species while *Pseudomonas*, *Streptococcus*, *Corynebacterium*, *Staphylococcus*, *Enterococcus*, *Bacillus* and *Escherichia* species were represented by 22, 16, 12, 11, 8, 7 and 6 different strains, respectively. However, in both metagenomes, *Acinetobacter johnsonii* XBB1 remained as the most abundant strain with a relative abundance of 39.03% and 31.23% in CM and H samples, respectively. The other predominant strains in CM metagenome were *Campylobacter mucosalis*, *Bacillus mycoides*, *Klebsiella pneumoniae* subsp. pneumoniae HS11286, *Leclercia adecarboxylata*, *Escherichia coli* str. K-12 substr. MG1655, *Escherichia coli* O157:H7 str. Sakai, *Escherichia coli* UMN026, *Escherichia coli* IAI39, *Staphylococ cusaureus* subsp. aureus NCTC 8325, *Staphylococcus xylosus*, *Bacillus subtilis* subsp. subtilis str. 168, *Mycobacterium* sp. Root 265 and *Macrococcus caseolyticus*. Importantly, this study demonstrated that 68.04% of the detected bacterial strains were exclusively found in CM milk metagenome, and among them *Pantoea dispersa* EGD-AAK13, *Klebsiella oxytoca*, *Kluyvera intermedia*, *Shewanella oneidensis* MR-1, *Kluyvera ascorbata* ATCC 33433, *Klebsiella aerogenes* KCTC 2190, *Kluyvera cryocrescens* NBRC 102467, *Acinetobacter pittii* PHEA-2, *Pseudomonas mendocina* ymp and *Acinetobacter gyllenbergii* NIPH 230 were the most predominant strains. Furthermore, most of these strains were previously unreported and possibly played an opportunistic role in the mammary gland pathogenesis (Supplementary Data 2; Table 5).

### CM-associated changes of archaea and viruses at the genus level

**Figure 5:**
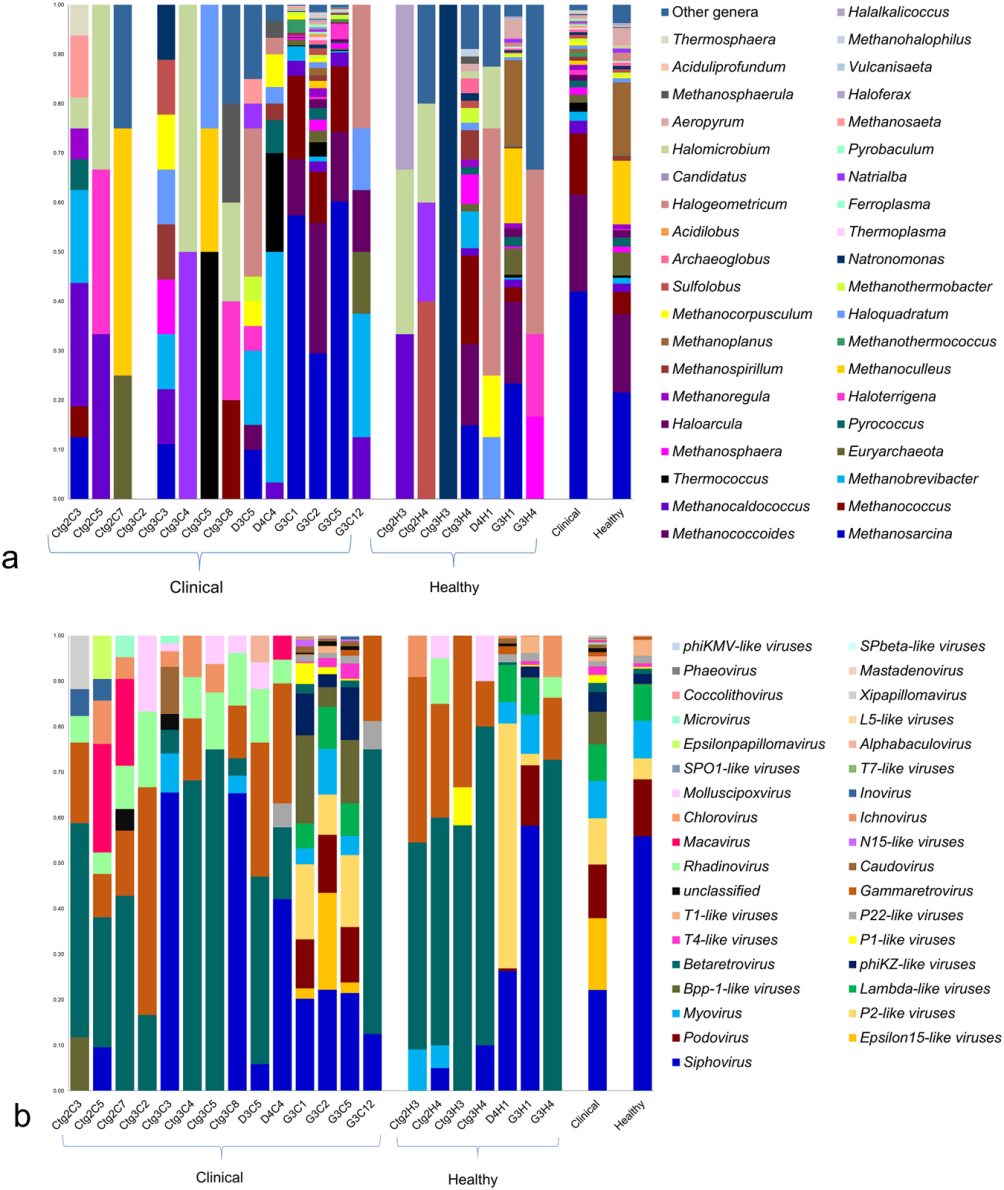
Taxonomic abundance of top 40 archaeal and viral genera in clinical mastitis (CM) and healthy (H) milk through MG-RAST (MR). (**a**) The relative abundance of 39 most abundant archaeal genera are sorted by descending order, with the remaining genera keeping into the ‘Other genera’. Archaeal genera are found in 20 samples, and absent in one clinical sample (Ctg3C2). (**b**) Taxonomic distribution of 35 viral genera detected in all of the 21 samples of clinical (CM) and healthy (H) milk metagenomes. The most abundant viral genera are sorted by descending order of the relative abundance. Each stacked bar plot represents the abundance of archaea and viruses in each sample of the corresponding category, where the last two bar plots depict overall relative abundance of archaeal and viral genera in both metagenome groups. Notable differences between the archaeal and viral populations are those where the taxon is abundant in clinical samples and effectively undetected in the healthy milk. Sample names: suffix ends with C refers to clinical (CM) and that ends with H refers to healthy (H) milk samples.

Another noteworthy finding of this study is the detection of archaeal (relative abundance 0.13%, p = 0.025) and viral (relative abundance 0.38%, p = 0.019) components of the microbiome both in CM and H milk samples (Fig. 5a,b; Supplementary Data 2). The CM metagenome was dominated by *Methanosarcina* (41.94%), *Methanococcoides* (19.58%), *Methanococcus* (12.30%), *Methanocaldococcus* (2.59%), *Methanobrevibacter* (1.85%), *Thermococcus* (1.79%) and *Methanosphaera* (1.53%) archaeal genera with a lower relative abundance (<0.05%) of the rest of the genera (Fig. 5a). Interestingly, none of the archaeal genera were detected in one CM sample (Ctg3C2). In contrast, *Methanoplanus* (14.69%), *Methanoculleus* (12.85%), *Euryarchaeota* (4.67%) and *Haloarcula* (1.50%) were the most abundant archaeal genera in H samples. The viral fraction of the current bovine milk microbiome was largely dominated by the members of the Caudovirales order, represented by the *Podoviridae*, *Siphoviridae* and *Myoviridae* families. The predominating viral genera found in CM were *Epsilon15-like viruses* (15.78%), *P2-like viruses* (10.12%), *Myovirus* (8.18%), *Lambda-like viruses* (8.06%), *Bpp-1-like viruses* (7.12%), *phiKZ-like viruses* (4.35%), *Betaretrovirus* (2.01%), *P1-like viruses* (1.79%) and *T4-like viruses* (1.79%). The H milk, however, had a relatively higher abundance of *Siphovirus* (55.85%), *Podovirus* (12.49%), *T1-like viruses* (3.44%) and *P22-like viruses* (1.71%) (Fig. 5b).

### Microbial metabolic functions associated with CM

**Figure 6:**
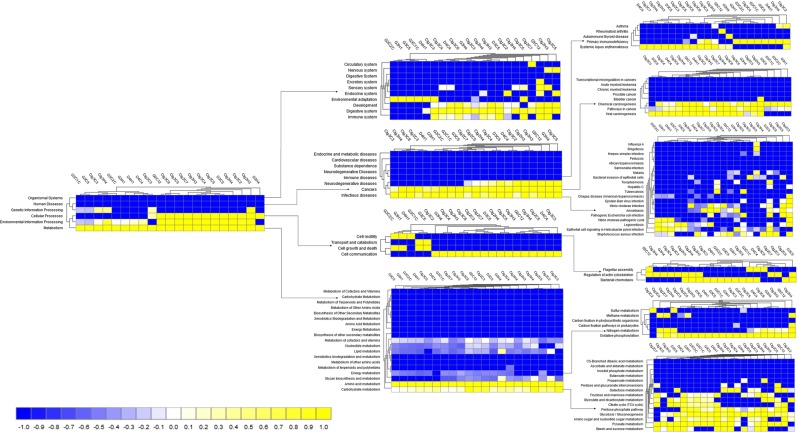
Projection of the clinical mastitis (CM) and healthy (H) milk metagenome onto KEGG pathways. The whole metagenome sequencing (WMS) reveals significant differences (*p* = 0.001) in functional microbial pathways. Heatmaps show the average relative abundance hierarchical clustering of the predicted KEGG Orthologs (KOs) functional pathways of the microbiome across all samples. The color bar at the bottom represents the relative abundance of putative genes. The color codes indicate the presence and completeness of each KEGG module, expressed as a value between -1 (low abundance) and 1 (high abundance). The yellow color indicates the more abundant patterns, whilst blue cells accounts for less abundant KOs in that particular sample. The color bar at the bottom represents the higher relative abundance of putative genes. Sample name: suffix ends with C refers to clinical mastitis (CM) and that ends with H refers to healthy (H) milk samples.

**Figure 7:**
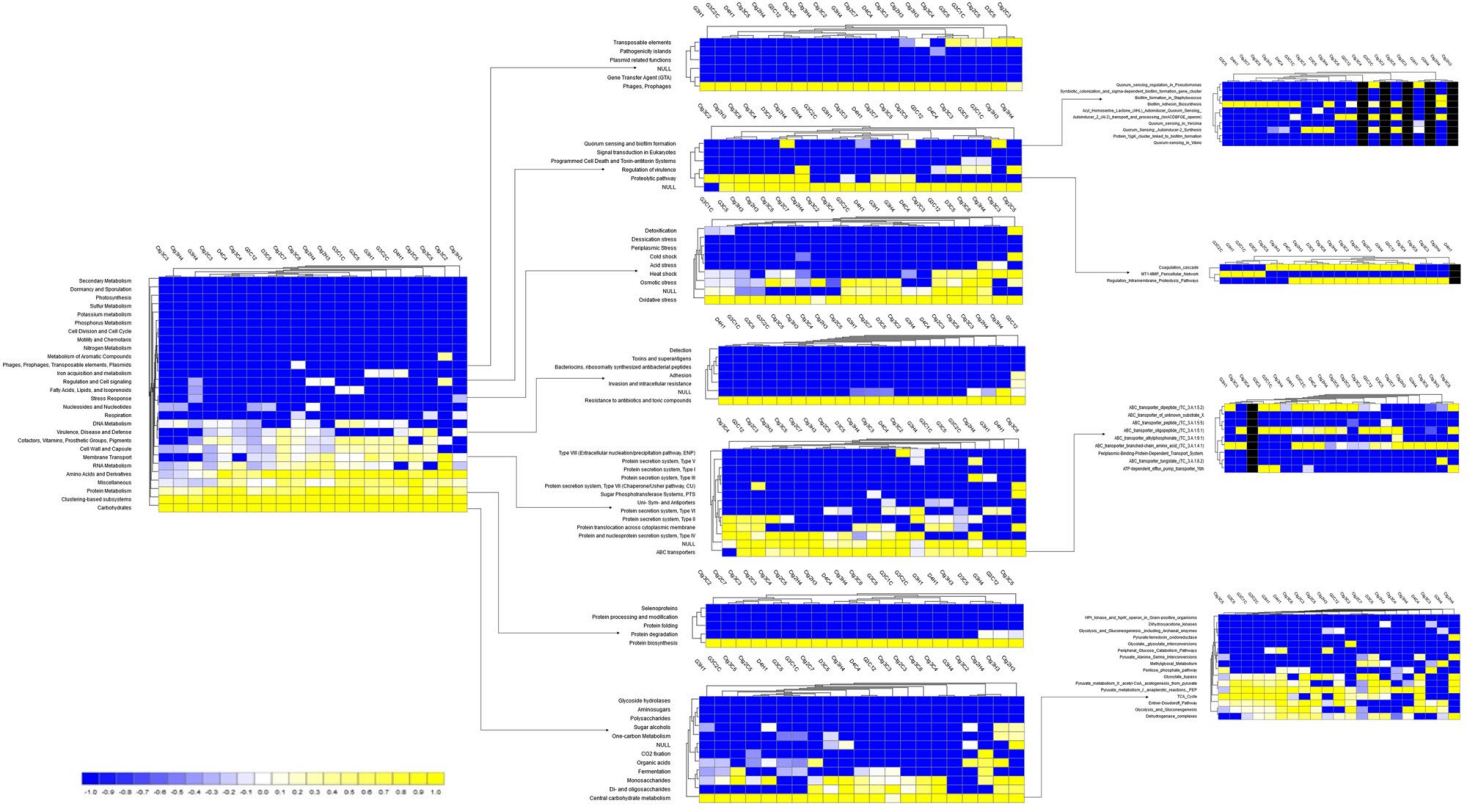
Functional annotation of the clinical mastitis (CM) and healthy (H) milk metagenome using different levels of SEED subsystem. Comparison of metagenomic profiles of CM and H milk microbiome at different levels of SEED subsystems (level 1–3). The selected subsystems showing significant (*p* < 0.05) differences between the two sample groups is shown. The less abundant subsystems in a given metagenome are shown in blue and subsystems that are more abundant are represented in yellow colors. The color codes indicated the presence and completeness of each subsystem module, expressed as a value between -1 (low abundance) and 1 (high abundance). The color bar at the bottom represents the higher relative abundance of putative genes. Sample name: suffix ends with C refers to clinical mastitis (CM) and that ends with H refers to healthy (H) milk samples.

MR simultaneously analyzed and compared the taxonomic composition and functional profiles of our metagenomic sequences in several ways. On average, the putative genes with known and unknown protein functions were 3.94% and 5.51%, respectively, suggesting that a large proportion of the genes encoding for different functional properties are yet unknown (Supplementary Data 1). By comparing the number of genes assigned to each KEGG pathway between the groups, we found a series of significant differences (*p* = 0.001) that lead to the functional divergence between the CM and H milk microbiome groups. The PCoA analysis of functional components showed significant differences between the CM and H samples indicating significant functional differences (*p* = 0.035) (Supplementary Fig. 4). In the comparative analysis, we found that genes associated with metabolism (central carbohydrate, amino acids, cofactors, vitamins, prosthetic groups and pigment), substrate dependence, clustering-based subsystems, cell motility (bacterial chemotaxis, flagellar assembly, invasion of epithelial cells), phases, prophages, transposable elements and plasmids, regulation and cell signaling, stress response, virulence, disease and defense, and immune and infectious diseases were significantly (*p* < 0.05) overrepresented and positively correlated with bovine CM (Figs 6 and 7; Supplementary Data 3).

Genes associated with citrate synthase (CS, *glt*A), fumarate hydratase class I (*fum*A, *fum*B), oxidative phosphorylation, bacterial translation, ribosome biogenesis and tRNA amino-acylation were significantly enriched in the metabolic pathways of CM associated microbiomes. The CM associated microbiome had significantly (*p* < 0.001) higher relative abundance (50.51%) of genes coding for benzoate degradation than the H milk biomes (36.41%). The CM samples had upregulation of genes for energy metabolism including carbon, sulfur, and methane metabolism compared to the H samples. The relative abundance of genes encoding ABC transporter (38.97%) and bacterial chemotaxis (68.61%) were significantly higher in CM microbes than those detected in H milk microbiomes (*p* < 0.005). Among the pathways in infectious diseases, genes coding for epithelial cell signaling, epithelial cells invasion, Legionellosis, *Vibrio cholerae* pathogenic cycle, *Staphylococcus aureus*, *Salmonella* and pathogenic *Escherichia coli* infection were most abundant in the CM metagenomes. Likewise, there was a predominant abundance of genes responsible for glutathione S-transferase (GST), breakpoint cluster region protein (BCR1), fumarate hydratase class II (*fum*C) and pyruvate kinase (pk) in different pathways causing mammary gland inflammation. The CM milk microbiomes had a significantly (*p* < 0.001) higher number of reads (64.29%) coding for severely combined immune deficient gene adenosine deaminase (ADA) compared to H milk microbes (28.58%) (Supplementary Fig. 5). Furthermore, sporulation related hypotheticals and CRISPR-associated proteins (*Cas*1, *Cas*2 and *Cas3)* remained higher in CM metagenomes compared to H milk microbes (Supplementary Data 3).

**Figure 8:**
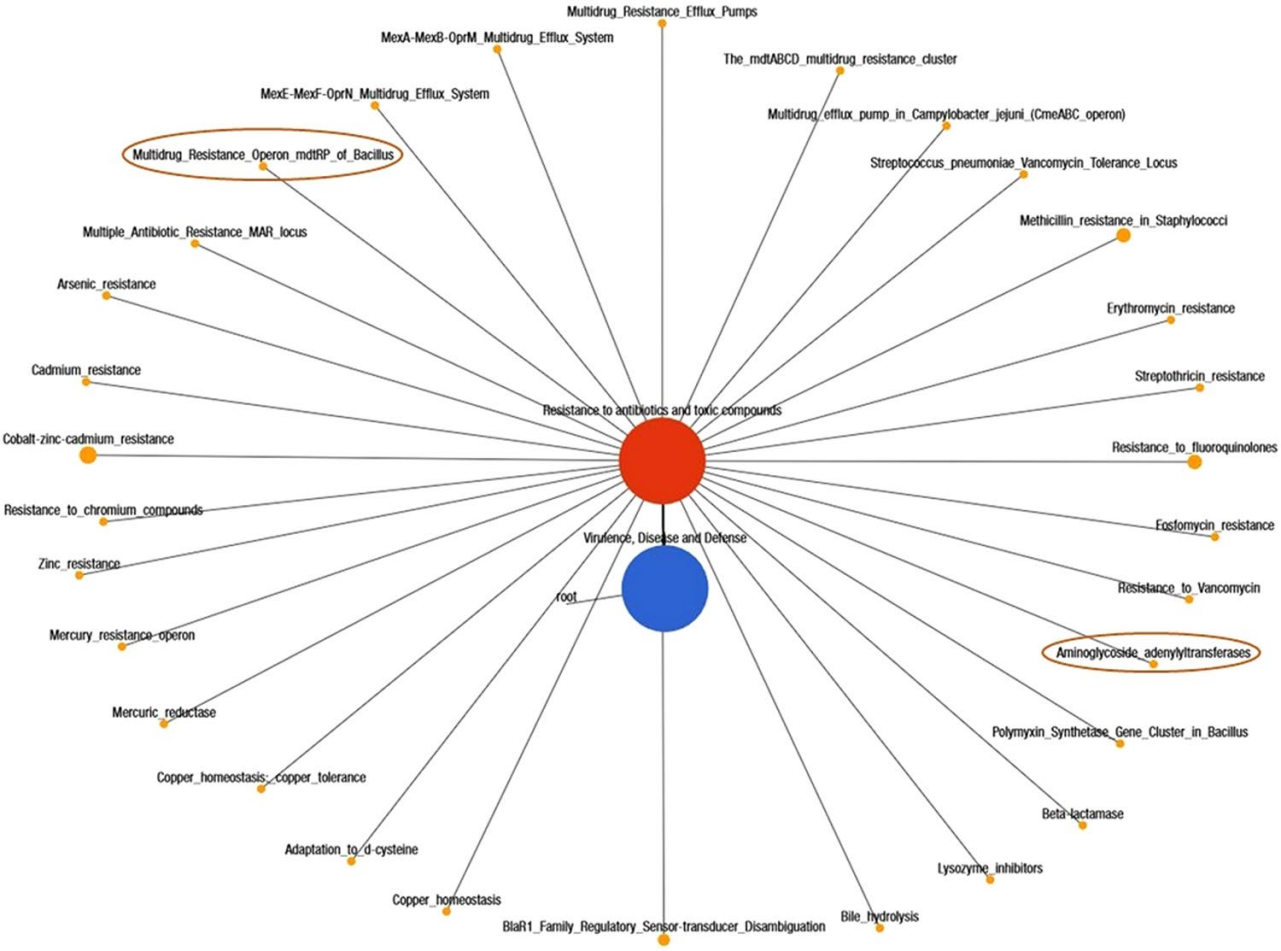
Networks showing distribution of the resistance to antibiotic and toxic compounds (RATC) genes in bovine milk metagenomes. A total of 6.45 million reads mapped to different levels of SEED subsystems in MR pipeline, of which 2.63% reads mapped against 30 and 28 different RATC genes in CM and H milk metagenomes, respectively. Black lines with yellow circles demarcate the distribution of the resistant genes according to their class across the both metagenomes. The diameter of the circles indicates the relative abundance of the respective genes in both clinical mastitis and healthy milk samples. The two differentially expressed genes (multidrug resistance to operon, *mdt*RP and aminoglycoside adenyltransferase) in CM milk metagenome are highlighted in deep yellow circles.

We found that the CM microbiome had significantly higher abundance of genes encoding for oxidative stress (36.46%), pathogenicity islands (10.13%), phage related transposable elements (19.48%), phage packaging machinery (6.37%), phage replication (6.70%) and phage regulatory gene expression (7.10%) compared to those of H milk biomes (*p* < 0.003). However, the phage lysogenic conversion related genes remained higher in abundance among the healthy milk microbes. A deeper look at microbial genes associated with regulation and cell signaling revealed that CM microbes had significantly higher expression of this gene compared to healthy milk microbiome (*p* = 0.001). Within this subsystem, genes coding for two-component regulatory system BarA-UvrY (*Sir*A; CM = 85.78% vs H = 67.41%), pericellular trafficking and cell invasion- the membrane type-1 matrix metalloproteinase (MT1-MMP; CM = 86.59% vs H = 73.80%), programmed cell death (CM = 55.00% vs H = 28.57%) and intra-membrane regulatory proteolytic pathway- endoplasmic reticulum chaperon *grp*78 (BiP; CM = 92.85% vs H = 71.42%) were predominantly found to be associated with the onset of bovine CM. We also identified novel associations of biofilm formation (BF) properties among the microbes identified in both metagenomes. The relative abundance of genes coding for protein *Yjg*K cluster linked to biofilm formation, biofilm PGA synthesis, deacetylase *Pga*B, N-glycosyltransferase *Pga*C and auxiliary protein *Pga*D were over-expressed in mastitis-causing pathogens (*p* = 0.035). In contrast, the genes coding for quorum sensing (QS) in particular to QS in *Yersinia*, *Pseudomonas* and *Vibrio* remained overexpressed in H milk metagenomes. Moreover, of the reads assigned to different levels of SEED subsystems (6.45 million), 2.63% mapped against 30 and 28 different resistance to antibiotic and toxic compounds (RATC) genes in CM and H milk metagenomes, respectively (Fig. 8; Supplementary Data 3). Among them, genes encoding multidrug resistance (efflux pumps, *mdt*ABCD cluster, *Cme*ABC operon), methicillin, vancomycin, and compounds (arsenic and chromium) resistance had two-fold higher relative abundances in CM microbiomes than H milk microbiomes. There was 5 to 7-fold overexpression of multidrug resistance to MAR locus and mercury resistance genes in CM microbes than in H milk organisms. In addition, CM-causing microorganisms harbored two additional resistance genes; multidrug resistance to operon (*mdt*RP) and aminoglycoside adenyltransferase (Supplementary Data 3).

## Discussion

Over the past decade, metagenomics has helped shed light on the ‘known unknown’ component of the milk microbiome and enabled insights into its taxonomic composition, dynamics, and importance to cows udder health homeostasis. Metagenomic deep sequencing (WMS) of bovine milk has uncovered previously overlooked microbial populations of high complexity with potential roles in regulation of overall microbiome composition and function, and in the onset, progression, and treatment strategies of bovine CM. Yet today, 16Sr RNA partial gene sequencing remains the dominant technique for characterizing milk microbiomes, and findings are limited to bacterial identification at the genus level^5,9,18^, though this method has serious inherent limitations^19^. However, little is known about the association of other microbes (archaea and viruses), microbiome shift and particularly functional changes. The noteworthy findings of the present WMS study are the taxonomic profiling of bacteria at both the species and/or strain-level, the possible association of the archaeal and viral fractions with bacterial mastitis, and the crosstalk between the identified microbiomes and their functional genomics in the association of bovine CM.

The findings generated from shotgun metagenomic data are much higher in taxonomic resolution and predicted protein functions and are consistent with previous 16S rRNA partial gene based studies^1,9,18^. The core bacteria associated with bovine CM such as *Acinetobacter*, *Pseudomonas*, *Klebsiella*, *Escherichia*, *Enterobacter*, *Staphylococcus*, *Streptococcus*, *Bacillus*, *Pantoea*, *Shewanella*, *Ralstonia* etc. remained consistent with the metagenomic data regardless of the analytic tool. Though CM milk samples had relatively higher taxonomic abundance, their abundance remained more inconsistent corroborating several recent findings^5,18,20^. To date, around 50 bacterial genera have been reported in bovine milk through 16Sr RNA-based targeted amplicon sequencing^1,9,18,21^, while our current WMS study detected 356 and 251 bacterial genera in CM and H milk, respectively indicating the increased discriminatory power of this cutting-edge technology in identifying taxa in the milk microbiome^10,16^. The observed increase in phylum-level signature of *Proteobacteria*, *Bacteroidetes*, *Firmicutes* and *Actinobacteria* in CM milk independent of quarter, parity, and breeds of the cows is mostly consistent with many of the previous studies^5,9,21^. Furthermore, the CM milk metagenome had an inclusion of 68.04% previously unreported bacterial species and/or strains, most of which are opportunistic in nature. Until now, no substantial information was available regarding the association of different strains of *Acinetobacter* with bovine mastitis^22^. In a recent study, association of *Acinetobacter* causing bubaline CM^7^ has been reported, supporting our present findings. The H milk metagenome had higher relative abundance of soil or environmental microbes (*Micromonospora*)^23^ and animal skin microbes (*Pseudomonas*)^24^, which can potentially act as opportunistic infection leading to disease. *Klebsiella pneumoniae* is an opportunistic environmental pathogen, and transmission of this bacterium might occur from contaminated feces and bedding materials^25^. The gut microbiome plays a key role in maintenance of nutrition, host defense, and immune development^26^, and we revealed a close association between the gut microbiome and milk microbes in the pathogenesis of bovine CM as also reported previously^27^. Additional support for this finding includes the potential existence of an endogenous entero-mammary pathway, through which gut bacteria migrate to the mammary gland, and this could explain the predominating presence of gut bacteria such as the phyla *Proteobacteria*, *Bacteroidetes*, *Firmicutes*, *Actinobacteria*, *Fusobacteria* and *Tenericutes*, with *Acinetobacter*, *Campylobacter*, *Bacillus*, *Enterobacter*, *Staphylococcus*, *Streptococcus* and *Kocuria* genera in CM milk^26,27^. These pathogens use very efficient strategies to evade host defenses in order to colonize and invade mammary tissues through adhesion^28^, thereby damaging host cells and fighting with cow immune systems to produce clinical and/or chronic mastitis^28–30^.

Our study marks an additional step towards identifying the significant co-occurrence of archaea and viruses with bacterial population in bovine milk. In comparison to bacteria, the relative abundance and diversity of archaea^31^ and viruses^32^ remain substantially lower. Currently, there is no extensive evidence supporting the role of archaea and viruses in the pathogenesis of bovine mastitis. However, these microbes mostly seize the opportunity during the pathophysiological changes in the mammary glands created by bacteria^33^. Thus, it is hypothesized that archaea might follow the exact mechanisms of bacterial pathogens producing bovine CM^31^. Most of the detected viral genera belonged to the order *Caudovirales* which consists of the three families of tailed bacterial viruses (bacteriophages) infecting bacteria and archaea. The host range of *Caudovirales* is very broad and includes all major bacterial phyla found in our samples: *Firmicutes*, *Bacteroidetes*, *Proteobacteria* and *Actinobacteria*. This corresponded with an increased relative abundance of these bacterial taxa in CM milk samples together with an overrepresentation of *Caudovirales* taxa compared with H milk samples^34^. In addition, we found a significant association of *Herpesvirales* (*Macavirus* and *Rhadinovirus* genera) with bovine CM^34,35^. Our current findings demonstrated that viruses neither cause bovine mastitis directly nor play a role in the initiation of the disease process, but later, when bacterial infection of the udder occurs, they replicate in the immune and epithelial cells of the udder and/or milk ducts and may act as a predisposing factor as well as a primary etiological agent for more severe and prolonged mastitis^36^.

The KEGG pathways and SEED subsystems of the MR pipeline uncovered significant differences in microbial metabolic functions in both CM and healthy samples^5,37^ as supported by several previous reports on mastitis in lactating cows^9^ and women^5^. The CM microbiome had significantly higher abundance of *Proteobacteria* and *Bacteroidetes*, which are well-known utilizers of milk oligosaccharides through one carbon metabolism^38^. Genes associated with the TCA cycle (*glt*A, *fum*A) and energy metabolism (oxidative phosphorylation) remained overexpressed in CM microbiomes, which might be associated with host-pathogen interactions during the progression of bovine mastitis^39^. Increased benzoate degradation by different strains of *Acinetobacter* and *Klebsiella* in CM metagenome through TCA cycle is thought to promote bacterial growth and virulence factors expressed during pathogenesis^40^. To elucidate the role of bacterial cell to cell communication in bovine mastitis, we found that genes coding for bacterial chemotaxis (*cheBR*,* motB*, *rbsB* and* tsr*) remained predominantly abundant in CM milk microbiomes suggesting their role in the early phase of mastitis for attachment to or entry into the udder tissues and virulence regulation^41^. The *p38* signaling pathway exerts its biological effects in the pathophysiology of bovine CM through several complex biologic processes including expression of many cytokines, transcription factors, cell surface receptors, enzymes and oxidative stress mediators^42,43^. The up-regulation of genes coding for programmed-cell death during host–pathogen interactions in CM is associated with increased secretion of bacterial toxins or pro-inflammatory mediators^44^. Biofilm formation can be a strain specific or genetically linked trait, representing a selective advantage in pathogenesis of mastitis. The relative overexpression of genes encoding the protein *Yjg*K cluster linked to biofilm formation and biofilm PGA synthesis in CM microbiomes is in accordance with several earlier reports^45^. Moreover, biofilm formation can also be harmful to host tissues since they can promote the phagocyte release of lysosomal enzymes, proliferation of reactive oxygen and nitrogen species, and transfer of antibiotic resistance^45^. The observed increased abundance of genes for primary immune diseases (e.g., adenosine deaminase) in CM pathogens is responsible for inhibition of T cell maturation and lymphocytic proliferation^46^, very low CD4 count^47^, cell-to-cell communication^47^ and therefore could be used as a selective marker for bovine CM diagnosis. CRISPR/*Cas* systems are present in both pathogenic and commensal organisms found in bovine milk and play critical roles during the pathogenesis of mastitis by evading the hosts defense system particularly under stress conditions^48^. The type III and IV secretion systems found on the pathogenicity islands of CM associated microbes are capable of producing immunosuppression in cows by delivering effector proteins^9,49^. Phages, which are the regulators of bacterial population, play important and diverse roles in all bacterial ecosystems^36^, but their precise impact on the milk microbiome is far from being understood. The relative overrepresentation of genes coding for phage-related transposable elements, phage packaging machinery, phage replication and phage regulatory gene expression in CM microbes may suggest that bacteriophages participate in the horizontal gene transfer among the members of bovine milk microbiomes and ultimately to mammary gland pathogens^34^.

Bovine milk microbiomes are a wide source of resistance to antibiotic and toxic compounds (RATC) genes and the pathogenic bacteria within this potential reservoir are becoming more resistant. The current metagenomic deep sequencing provides a wealth of information not only on RATC genes, but on the entire gene content thereby enabling the identification of the community composition and metabolic profile. We found that all of the samples in both metagenomes harbored RATC genes (2.63%) indicating their wide and indiscriminate use in Bangladeshi dairy farms. However, most of the resistant genes in RATC functional groups remained predominantly higher in CM milk microbes. While our knowledge of the uncontrolled spread of antibiotics resistant genes in bovine mastitis pathogens^50^ is increasing, information on heavy metal resistance is not yet available. This worrisome trend in increasing RATC against mastitis pathogens has become a major concern for the dairy farmers of Bangladesh, given the seriousness of such problems; effective therapies using alternative medicines are needed for successful prevention and control of bovine mastitis.

## Conclusions

In this study, the metagenomics of milk samples from bovine with clinical mastitis (CM) and healthy (H) controls clearly show that the microbiome composition in CM milk samples are significantly different from H milk. Furthermore, some of the detected microbes (bacteria, 68.04%, archaea, 31.82% and viruses, 40.00%) are solely found only in CM samples. The co-relations of the microbiome composition and functional metagenomics in the progression of clinical mastitis are also evidenced by abundance differences in metabolic pathways related to bacterial colonization, proliferation, chemotaxis and invasion, immune-diseases, oxidative stress, regulation and cell signaling, antimicrobial resistant genes, biofilm formation, phage and prophases etc. between CM and H samples. The presence of human pathogens including *Escherichia coli* O157:H7 str. Sakai, *Salmonella enterica* subsp. enterica serovar Typhi str. CT18, *Salmonella enterica* subsp. enterica serovar Typhimurium str. LT2, *Bacillus cereus* ATCC 14579 etc. in bovine milk and RATC genes in milk microbiome are serious concerns for public and animal health since diseased animals are improperly handled in Bangladesh. Because of the limitations we faced with fewer samples, it would be interesting to conduct similar trials using a larger sample size with a different animal population (breed, parity, body condition, lactation) and matrices prior to undertaking a metagenomics sequencing venture to elucidate the progression of the disease. Furthermore, such studies would also be enhanced by the inclusion of gut microbiome sampling in addition to the milk samples for direct testing of microbial transfer across this axis.

## Methods

### Study population and sampling

Details of study population and collected samples are presented in Supplementary Table 1. A total of 21 milk samples (14, CM and 7, H) from 21 lactating crossbred cows at their early stage of lactation (within 10–40 days of calving) were collected from three districts of Bangladesh (Chattogram = 12, Dhaka = 3, Gazipur = 6). Cows were diagnosed with CM using the California mastitis test^51^. Two CM and one H milk samples were collected from the same farm. Approximately 15–20 ml of milk from each cow was collected in a sterile falcon tube during the morning milking (8.0–10.0 am) with an emphasis on pre-sampling disinfection of teat-ends and hygiene during sampling^1,51^. The samples were kept in an ice box (at 4 °C temp) immediately after collection, transported to the laboratory following similar transport protocols, and stored at −20 °C until DNA extraction.

### DNA extraction and sequencing

Genomic DNA (gDNA) was extracted by an automated DNA extraction platform (Promega, UK) following previously described protocols^5,17^. DNA quantity and purity was determined with NanoDrop (ThermoFisher, USA) by measuring 260/280 absorbance ratios. Sequencing libraries were prepared with Nextera XT DNA Library Preparation Kit^52^ according to the manufacturer’s instructions and paired-end (2 × 150 bp) sequencing was performed on a NextSeq 500 machine (Illumina Inc., USA) at the George Washington University Genomics Core facility. Our metagenomic DNA yielded 483.38 million reads with an average of 23.01 million (maximum = 35.10 million, minimum = 6.77 million) reads per sample (Supplementary Data 1).

### Sequence reads preprocessing

The resulting FASTQ files were concatenated and filtered through BBDuk^14^ (with options k = 21, mink = 6, ktrim = r, ftm = 5, qtrim = rl, trimq = 20, minlen = 30, overwrite = true) to remove Illumina adapters, known Illumina artifacts, and phiX. Any sequence below these thresholds or reads containing more than one ‘N’ were discarded. On average, 20.16 million reads per sample (maximum = 32.33 million, minimum = 4.71 million) passed the quality control step (Supplementary Data 1).

### Microbiome community analysis

We analyzed the WMS data using mapping-based and assembly-based hybrid methods of PathoScope 2.0 (PS)^53^ and MG-RAST 4.0 (MR)^8, respectively^. In PS analysis, a ‘target’ genome library was constructed containing all bacterial and archaeal sequences from the NCBI Database (https://en.wikipedia.org/wiki/National_Center_for_Biotechnology_Information) using the PathoLib module. The reads were then aligned against the target libraries using the very sensitive Bowtie2 algorithm^16,17^ and filtered to remove the reads aligned with the cattle genome (bosTau8) and human genome (hg38) as implemented in PathoMap (-very-sensitive-local -k 100–score-min L, 20, 1.0). Finally, the PathoID^54^ module was applied to obtain accurate read counts for downstream analysis. In these samples, an average of 12.90 million aligned reads per sample mapped to the target reference genome libraries (96.24%) after filtering the cow and human genome (Supplementary Data 1). The raw sequences were simultaneously uploaded in MR server (release 4.0) with proper embedded metadata and were subjected to the quality filter containing dereplication and removal of host DNA by screening^55^ for taxonomic and functional assignment.

### Diversity analysis

Alpha diversity (diversity within samples) was estimated using the Shannon index for both PS and MR reads. To test beta diversity (differences in the organismal structure) of the milk microbiome, a principal coordinate analysis (PCoA) was performed based on weighted-UniFrac distances (for PS data) through Phyloseq R^56^ and Bray-Curtis dissimilarity matrix^57^ for MR data. In addition, non-metric multidimensional scaling (NMDS) on PS data was also used for beta diversity^58^ analysis between the sample groups^59^. Taxonomic abundance was determined by applying the “Best Hit Classification” option using the NCBI database as a reference with the following settings: maximum e-value of 1 × 10^−30^, minimum identity of 95% for bacteria, 60% for archaea and viruses and a minimum alignment length of 20 as the set parameters. The phylogenetic origin of the metagenomic sequences was projected against the NCBI taxonomic tree and determined by the lowest common ancestor (LCA) with the same cutoff mentioned above. Two phylogenetic trees consisting of 363 and 146 bacterial strains in CM and H metagenomes, respectively, with >80% taxonomic identity were constructed using the neighbor-joining method in Clustal W (version 2.1)^60^ and visualized with FigTree (version 1.5.1)^14^.

### Statistical analysis

The characteristics of cows with and without CM were compared using Fisher’s exact test for categorical variables and Mann-Whitney U test for quantitative variables^5,7–9^. The Shapiro-Wilk test was used to check normality of the data and the non-parametric test Kruskal-Wallis rank sum test was used to evaluate differences in the relative percent abundance of taxa in CM and H groups. The statistical analyses for the MR data were initially performed by embedded calls to statistical tests in the pipeline and validated further using SPSS (SPSS, Version 23.0, IBM Corp., NY USA) using above mentioned tests. For the functional abundance profiling, the statistical tests were applied at different KEGG and SEED subsystem levels in the MR pipeline. Differences between the pipelines were evaluated using ANOVA and the Friedman rank sum test. A significance level of alpha = 0.05 was used for all tests^8^.

## Supplementary information

Supplementary Figures with legends

Supplementary Table 1

Supplementary Data 1

Supplementary Data 2

Supplementary Data 3

Supplementary Table 2.

Supplementary Table 3.

Supplementary Table 4.

Supplementary Table 5.

## Data Availability

The sequence data reported in this paper have been deposited in the NCBI database (BioProject PRJNA529353) and are available from the corresponding author upon reasonable request.
