# Supplementary material for: Metagenomic deep sequencing reveals association of microbiome signature with functional biases in bovine mastitis
Source: Sci Rep. 2019 Sep 19;9:13536. doi: 10.1038/s41598-019-49468-4 (PMC6753130; doi:10.1038/s41598-019-49468-4)
Supplement: Supplementary file 6 — Supplementary Table 2. [file 41598_2019_49468_MOESM6_ESM.docx]

**Metagenomic deep sequencing reveals association of microbiome signature with functional biases in bovine mastitis**

M. Nazmul Hoque, Arif Istiaq, Rebecca A. Clement, Munawar Sultana, Keith A. Crandall, AMAM Zonaed Siddiki, M. Anwar Hossain

**Supplementary Table 2:** Taxonomic distribution of bacteria in clinical mastitis (CM) and healthy (H) milk samples by PathoScope (PS) and MG-RAST (MR) analysis (NA, Not detected by the pipeline).

| **Taxonomic ranks** | **PS** | | **MR** | |
| --- | --- | --- | --- | --- |
|  | CM | H | CM | H |
| Phylum | 8 | 4 | 18 | 12 |
| Class | 17 | 9 | 30 | 22 |
| Order | 44 | 33 | 73 | 53 |
| Family | 90 | 41 | 163 | 124 |
| Genus | 116 | 66 | 359 | 253 |
| Species | 363 | 146 | NA | NA |
